# Supplementary material for: Candidate Genes and Pathways in Cervical Cancer: A Systematic Review and Integrated Bioinformatic Analysis
Source: Cancers (Basel). 2023 Jan 30;15(3):853. doi: 10.3390/cancers15030853 (PMC9913780; doi:10.3390/cancers15030853)
Supplement: Supplementary file 1 [file cancers-15-00853-s001.zip › cancers-2138368-supplementary.pdf]

cancers

MDPI

*Supplementary Materials*

# Candidate Genes and Pathways in Cervical Cancer: A Systematic Review and Integrated Bioinformatic Analysis

**Marjanu Hikmah Elias, Srijit Das and Nazefah Abdul Hamid**

**Table S1.** Quality assessment of the studies included in the systematic review.

| No. | Author, Year | Questions assessing – case control studies | | | | | | | | | | Yes (%) |
|---|---|---|---|---|---|---|---|---|---|---|---|---|
| | | 1 | 2 | 3 | 4 | 5 | 6 | 7 | 8 | 9 | 10 | |
| 1 | Annapurna et al., 2021 [19] | Y | Y | Y | Y | Y | N | N | Y | Y | Y | 80 |
| 2 | Kim et al., 2013 [20] | Y | Y | U | Y | Y | N | N | Y | Y | Y | 70 |
| 3 | Rajkumar et al., 2011 [21] | N | Y | U | Y | Y | N | N | Y | Y | Y | 60 |
| 4 | Wong et al., 2006 [24] | Y | Y | Y | Y | Y | N | N | Y | Y | Y | 80 |
| | | **Questions assessing – cross sectional studies** | | | | | | | | | | |
| 1 | Miyatake et al., 2007 [22] | Y | Y | Y | Y | N | N | Y | Y | | | 75 |
| 2 | Gius et al., 2007 [23] | Y | N | Y | Y | N | N | Y | Y | | | 63 |

Y=Yes; N=No; U=Unclear.

Case control studies assessment

Questions:

1. Were the groups comparable other than the presence of disease in cases or the absence of disease in controls?
2. Were cases and controls matched appropriately?
3. Were the same criteria used for identification of cases and controls?
4. Was exposure measured in a standard, valid and reliable way?
5. Was exposure measured in the same way for cases and controls?
6. Were confounding factors identified?
7. Were strategies to deal with confounding factors stated?
8. Were outcomes assessed in a standard, valid and reliable way for cases and controls?
9. Was the exposure period of interest long enough to be meaningful?
10. Was appropriate statistical analysis used?

Cross sectional studies assessment

Questions:

1. Were the criteria for inclusion in the sample clearly defined?
2. Were the study subjects and the setting described in detail?
3. Was the exposure measured in a valid and reliable way?
4. Were objective, standard criteria used for measurement of the condition?
5. Were confounding factors identified?
6. Were strategies to deal with confounding factors stated?
7. Were the outcomes measured in a valid and reliable way?
8. Was appropriate statistical analysis used?

**Table S2.** The common differentially expressed genes in at least two studies.

| Study ID | Common DEGs |
|---|---|
| Annapurna, Gius, Rajkumar, Wong [19,21,23,24] | *CDH3, CDKN2A* |

| | |
|---|---|
| Annapurna, Gius, Kim [19,20,23] | *BST2* |
| Annapurna, Gius, Wong [19,23,24] | *PLSCR1* |
| Gius, Rajkumar, Wong [21,23,24] | *SPINK5, PLOD2* |
| Annapurna, Kim [19,20] | *SFRP4, STAT1, FOS, CXCL12, POLR2H* |
| Annapurna, Rajkumar [19,21] | *SMC4, CKS1B* |
| Annapurna, Gius [19,23] | *EGR1, KLF4, ESR1, COL6A3, FOSB* |
| Annapurna, Wong [19,24] | *SPP1, DSG1, ALOX12* |
| Kim, Rajkumar [20,21] | *CD36, LAMB3, MCM4, MCM6* |
| Kim, Miyatake [20,22] | *PTGS2* |
| Gius, Kim [20,23] | *MNAT1, AK3, CHRNA5, DNAJA3, CD151, IER3, ITGAV, IFNAR1, CBX1, RAD51C, ELL, SGK, PPP1R12B, HNRPC, ISGF3G, IFI30, FBLN2, WISP1, CLCN3, IFITM1, COL1A2* |
| Kim, Wong [20,24] | *IFI16, IL18, GYS2, RRM2, ESR2* |
| Gius, Rajkumar [21,23] | *CDC25B, MMP3, CCNB2, CSTB* |
| Rajkumar, Wong [21,24] | *KRT10, SLURP1* |
| Miyatake, Wong [22,24] | *KRT1* |
| Gius, Wong [23,24] | *CRYAB, EMP1, PPL, VEGF, MCM2, IL1RN, KRT7, KLK11, CDA* |
